# Supplementary material for: Exploring Fungicide Sensitivity in Soybean Stem Blight Pathogen Diaporthe longicolla, Emphasizing Genetic Variability Impact on Response to SDHI Fungicides Fluopyram and Pydiflumetofen
Source: J Fungi (Basel). 2025 Apr 8;11(4):292. doi: 10.3390/jof11040292 (PMC12028546; doi:10.3390/jof11040292)
Supplement: Supplementary file 1 [file jof-11-00292-s001.zip › Supplemental Materials (clean version).pdf]

SUPPLEMENTAL MATERIALS

Table S1 *Diaporthe* species isolates obtained from soybeans in Heilongjiang, China, in 2021 and 2022.

Location (city, province)	Designation
Heihe, Coastal river manchu	01
Aihui, Waisandagou	02
Qike, Tuanqing	03
Aihui	04
Heihe, Sunke Farm Rural Road 307	05
Sunwu, Coastal river manchu-2	06
Sunwu, Qike	07
Heihe, Wudalianchi, Jianshe	08
Lianchi, Xinfa, Yingbin Road	09
Wudalianchi, Xinfa, Yingbin Road-2	10
Wudalianchi, Xinfa, Yingbin Road-3	11
Heihe, Beian, Dongsheng	12
Heihe, Beian, Erjing (Menghu43)	13
Wudalianchi, Xinfa, Yingbin Road-4	14

Table S2 The primers used to identify *Diaporthe longicalla*.

Primer	Sequence	Gene	Annealing temperature
ITS4	5’-TCCTCCGCTTATTGATATGC-3’	ITS	55℃
ITS5	5’-GGAAGTAAAAGTCGTAACAAGG-3’		
BT2a	5’-GGTAACCAAATCGGTGCTGCTTTC-3’	*β-tub*	58℃
BT2b	5’-ACCCTCAGTGTAGTGACCCTTGGC-3’		
EF1-728F	5’-CATCGAGAAGTTCGAGAAGG-3’	*EF1-α*	58℃
EF1-986R	5’-TACTTGAAGGAACCCTTACC-3’		
CAL-228F	5’-GAGTTCAAGGAGGCCTTCTCCC-3’	*CAL*	55℃
CAL-737R	5’-CATCTTCTGGCCATCATGG-3’		

Table S3 Collection information of standard strains employed to develop the multigene phylogenetic tree of *Diaporthe* isolates.

Species	GenBank No.[1]			
	ITS	*CAL*	*TEF*	*BT*
D. longicolla	KJ590728	KJ612124	KJ590767	KJ610883
D. sojae	KJ590719	KJ612116	KJ590762	KJ610875
D. unshiuensis	KJ490587	N/A	KJ490466	KJ490408
D. aspalathi	KC343036	KC343278	KC343762	KC344004
D. acaciigena	KC343005	KC343247	KC343731	KC343973
D. alleghaniensis	KC343007	KC343249	KC343733	KC343975
D. terebinthifolii	KC343216	KC343458	KC343942	KC344184
D. amygdali	KC343020	KC343262	KC343746	KC343988
D. arengae	KC343034	KC343276	KC343760	KC344002
D. bicincta	KC343134	KC343376	KC343860	KC344102
D. bohemiae	MG281015	MG281710	MG281536	MG281188
D. camptothecicola	KY203726	KY228877	KY228887	KY228893
D. celastrina	KC343047	KC343289	KC343773	KC344015
D. citri	KC843311	KC843157	KC843071	KC843187
D. endophytica	KC343065	KC343307	KC343791	KC344033
D. helinathi	KC343115	JX197454	GQ250308	KC344083
D. hongkonggensis	KC343119	KC343361	KC343845	KC344087
D. limonicola	MF418422	MF418256	MF418501	MF418582
D. melitensis	MF418424	MF418258	MF418503	MF418584
D. perjuncta	KC343172	KC343414	KC343898	KC344140
D. schini	KC343191	KC343433	KC343917	KC344159
D. rudis	KC343234	KC343476	KC343960	KC344202
Diaporthella corylina	KC343004	KC343246	KC343730	KC343972

[1] ITS: internal transcribed spacers 1 and 2 together with 5.8S nrDNA; *CAL*: partial calmodulin gene. Sequences generated in this study are indicated in italics; *TEF*: partial translation elongation factor 1-α gene; *BT*: partial beta-tubulin gene.

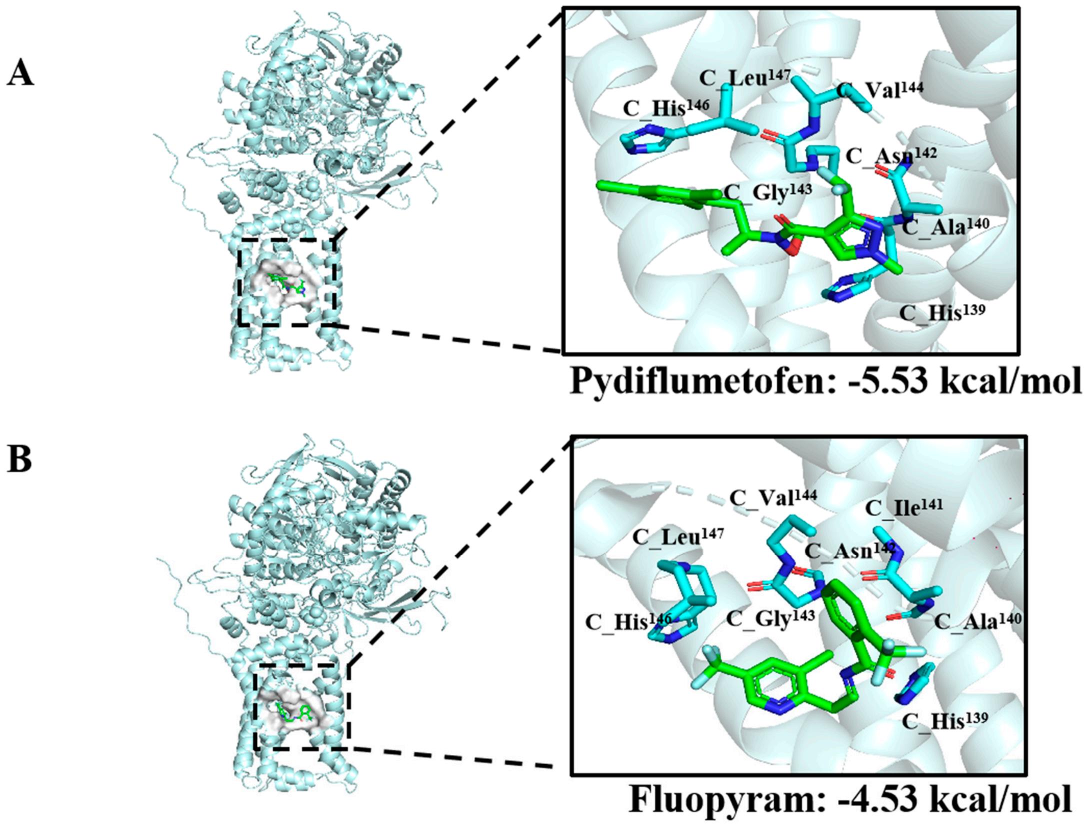

Figure S1 Molecular docking of pydiflumetofen and fluopyram into the SdhC subunit of *Diaporthe longicolla*. A) The binding energy and module of pydiflumetofen into the SdhC protein of *D. longicolla*, the residues where pydiflumetofen and SdhC protein bind are indicated in black font, and the binding free energy is -5.53 kcal/mol. **B)** The binding energy and module of fluopyram into the SdhC protein of *D. longicolla*. the residues where fluopyram and SdhC protein bind are indicated in black font, and the binding free energy is -4.53 kcal/mol.
